# Impact of Clerkship Length and Sequence on NBME Subject Exam Performance

**DOI:** 10.1007/s40670-025-02305-y

**Published:** 2025-02-17

**Authors:** Megan Vaughan, Kory A. Johnson, Christina R. Bergin

**Affiliations:** The University of Arizona College of Medicine – Phoenix, 475 N. 5th St., Phoenix, AZ 85004 USA

**Keywords:** Clerkship length, Medical education, NBME, Subject exams, Curriculum reform

## Abstract

**Purpose:**

Core clerkships are foundational learning experiences, yet variability in duration exists across medical schools. Many institutions adjust core clinical experiences as part of curricular modifications to meet evolving needs in undergraduate medical education. We investigated if shortened Internal Medicine (IM) and Surgery clerkship lengths or if clerkship sequence within the academic year would have any impact on NBME subject exam scores.

**Methods:**

We examined four cohorts of third-year medical students from academic years 2017–2018, 2018–2019, 2019–2020, and 2021–2022. Individual student NBME subject exam data were compared, controlling for MCAT score and clerkship block sequence within the academic year.

**Results:**

We found no statistically significant differences in IM or Surgery NBME subject exam scores between the traditional clerkship length (2017–2019) and shortened clerkship length (2019–2022) groups. Mixed-effect regression analyses that included MCAT and block sequence as additional covariates confirmed there were no statistically significant differences in IM or Surgery exam scores between groups. Despite no change in length, the Psychiatry (*p* = 0.012) and Pediatrics (*p* = 0.036) clerkships had increased scores post-intervention on the mixed-effects model. MCAT scores were predictive of overall NBME scores on both ANCOVA and regression analyses (*p*-values ranging < 0.001 to 0.01). Finally, taking a clerkship later in the academic year was associated with increased NBME scores across all subjects (*p* < 0.001).

**Conclusions:**

Shortened clerkship length is not associated with lower performance on NBME subject exams. Clerkship sequence later in the academic year is associated with higher scores. Curricular reform resulting in reduced core clerkship duration may be undertaken without adverse impact on medical knowledge.

**Supplementary Information:**

The online version contains supplementary material available at 10.1007/s40670-025-02305-y.

## Introduction

The goals of the core clerkships in medical school are to allow students time to perfect their skills in the core competencies, to foster supervised encounters in which students learn to apply medical knowledge to clinical scenarios, and to offer students hands-on experiences that provide a comprehensive knowledge of core medical disciplines [1]. The exact content of these clerkships and the amount of time allocated to specific skills within clerkships are variable across medical schools. In 2016, the National Board of Medical Examiners contacted the Executive Chief Proctors of 278 medical schools to acquire information pertaining to the format, length, and timing of clinical clerkships at each institution. They found that Emergency Medicine, Advanced Emergency Medicine, and Advanced Internal Medicine were universally maintained as a traditional curriculum, while Ambulatory Medicine was most likely to have an alternative format. Such alternative curricula included longitudinal rotations, problem-based learning, and combination traditional/longitudinal curricula lasting under 3 months. The length of individual clerkships across different institutions also varied significantly, with Surgery and Emergency Medicine being the most consistent across institutions at 8 weeks and 4 weeks, respectively. Ambulatory Medicine clerkships ranged from 4 to 16 weeks, Family Medicine from 2 to 20 + weeks, and Pediatrics from 1 to 16 weeks [2]. Despite nearly 100% of surveyed schools utilizing the NBME Subject Examinations as a metric for students to pass their clerkships, the content, length, and structure of these clerkships follow no set standard.

Studies examining the individual impact of altered clerkship length are limited. The ones that do exist demonstrate mixed results, and many also include changes to other clerkship components that occurred simultaneously with the adjustment in duration. Monrad et al. conducted a study in 2018 examining the effect of decreasing the length of all clerkships by 25% during the 2016–2017 academic year at the University of Michigan Medical School and found that student perceptions of clerkship quality improved [3]. Regarding objective measures of student performance, the study found no significant differences in performance on the clinical subject exams across all NBME subjects as compared to previous cohorts of students. Interestingly, these findings are in contrast with those from previous studies, which demonstrated a significant reduction in NBME subject exam scores when clerkship duration is shortened [4–8]. For instance, studies have shown that longer Internal Medicine [4], Surgery [5], Psychiatry [6, 7], and Obstetrics/Gynecology [8] rotations correlate with higher subject exam scores in their respective exams. Monrad et al. note that this discrepancy in findings may stem from a difference in the intervention and outcome analysis — they examined the impact of a shortened clerkship curriculum as a whole (multiple clerkship length reductions), while previous studies investigated the effect of a single abbreviated clerkship on its corresponding individual exam. A clear understanding of the impact of shortened clerkship length is further hindered by studies demonstrating that reduced length of clerkships may be a secondary factor on student performance when additional metrics are considered, for example — the timing of the clerkship in relation to other clerkships and within the academic year, other concurrent curricular revisions, and variable pre-clerkship and intra-clerkship clinical experiences, such as incorporating more active learning time [4, 9–14].

As the University of Arizona College of Medicine – Phoenix made several adjustments to the clinical curriculum beginning in the 2019–2020 academic year, it was vital for us to understand the impact of these changes on student medical knowledge. Notably, the Internal Medicine and Surgery clerkships were each shortened from 12 to 8 weeks without any adjustment to the preclinical curriculum or to the duration of other clerkship lengths. The rationale for shortening these two clerkships was to allow students an elective rotation in the third year, providing individualized learning opportunities tailored to their specific career interests. In addition to this 4-week elective, the Neurology clerkship (4 weeks) was moved from the fourth year into the third year to allow exposure to this specialty before the USMLE Step 2 Clinical Knowledge (CK) exam was taken. For a more detailed description of curriculum changes, please see Appendix 1.

The purpose of this study was to test two hypotheses: (1) the length of a clerkship does not significantly impact student performance on the associated NBME subject exam, and (2) the timing of a specific clerkship within the academic year’s sequence of core clerkships does impact student performance on the associated subject exam. We used NBME subject examination performance as a surrogate marker for medical and clinical knowledge. While other metrics of student performance exist (such as clinical performance ratings and final clerkship grades), these methods involve a certain level of subjectivity on the part of the grader. Utilizing the standardized exam was the best way to remove potential bias, as well as to increase generalizability outside our single institution.

This study helps fill a gap in current literature on this topic by evaluating the impact of reduction in length of two required clerkships without other major curricular changes, while also analyzing the impact of specific clerkship timing within the academic year and controlling for potential confounding variables, such as changes in medical school or hospital staff, changes to clinical didactics, and individual student test-taking skills.

## Methods

### Setting and Participant Selection

University of Arizona College of Medicine – Phoenix is a 4-year allopathic school with an abbreviated preclinical curriculum and entry into clerkships 3 months earlier than students at many other medical schools. Traditionally, the Year 3 curriculum comprised six core clerkships — two of which were 12 weeks in length (Internal Medicine and Surgery) and four of which were 6 weeks in length (Obstetrics and Gynecology (OB/GYN); Family, Community, and Preventive Medicine (Family Medicine or FM); Pediatrics; and Psychiatry). Core clerkships in Neurology and Emergency Medicine occurred in Year 4; each was 4 weeks long. Beginning in the 2019–2020 academic year, the Internal Medicine (IM) and Surgery clerkships were shortened to 8 weeks in order to permit movement of the 4-week Neurology clerkship and one 4-week elective from year 4 into year 3. These curricular changes were made to allow earlier individual career exploration through an elective and to provide better preparation for USMLE Step 2 CK through earlier Neurology exposure. To accommodate the reduction in IM and Surgery clerkship length, time within ambulatory IM and surgical subspecialties was reduced, with the option for students to obtain additional experience in these areas through year 4 electives. There were no additional changes to the structure of the core clerkship curricula. A longitudinal patient care course spanning years 3 and 4 similarly remained unchanged in the new clinical curriculum. The Emergency Medicine (EM) clerkship remained in year 4 without change.

All medical students at the University of Arizona College of Medicine – Phoenix who completed a clinical clerkship and NBME subject examination between the periods of 2017–2018, 2018–2019, 2019–2020, and 2021–2022 were included as participants, provided they had signed consent to participate in medical education research under the Research Office for Medical Education (ROME) Umbrella IRB for medical student data at our institution. Students completing core clerkships during academic year 2020–2021 were excluded because of significant pandemic-related alteration in clerkship length and curricula during that year. Data from the six core clerkships occurring in year 3 (FM, IM, OB/GYN, Pediatrics, Psychiatry, and Surgery) were included. EM and Neurology clerkship data were excluded because these clerkships were not taken consistently in the third year, and more clinical experience was considered a confounding factor. All research conducted within this study was approved under the ROME umbrella IRB and the Committee for Assessment and Evaluation of Submission Approval for Research (CAESAR) governed by ROME.

### Tested Hypotheses

The first hypothesis tested in the study is that reducing clerkship length does not impact student performance on the associated NBME subject exam. This metric, rather than clinical performance ratings or final clerkship grades, was chosen to avoid potential bias introduced by subjective rating patterns of individual faculty or by changes in faculty across academic years. Students from the traditional longer clerkship cohorts (academic years 2017–2018 and 2018–2019) served as the control or pre-intervention group. Students from the 2019–2020 and 2021–2022 cohorts were in the experimental or post-intervention group, with the IM and Surgery clerkship lengths each shortened from 12 to 8 weeks for this group. Other clerkship exam scores (OB/GYN, FM, Pediatrics, and Psychiatry) within these four cohorts of students served as internal controls, as their lengths remained the same.

We also tested a second hypothesis: the timing of an individual clerkship within the academic year’s sequence of core clerkships does impact student performance on the corresponding NBME subject exam. When testing this hypothesis, the IM, Surgery, Pediatrics, FM, OB/GYN, and Psychiatry subject exam scores were included for all four cohorts of students. In addition, individual student MCAT exam performance was examined as a potential confounding variable (data included for all four cohorts of students).

### Statistical Analysis

Information was extracted pertaining to NBME subject exam scores, MCAT performance, and the sequence of NBME subject exams taken for four cohorts of medical students at the University of Arizona College of Medicine – Phoenix. Descriptive statistics (namely, mean and standard deviation for continuous measures, and frequency and percentage for nominal measures) were calculated for these extracted measures. Differences across cohorts in these measures were assessed via one-way ANOVAs for continuous measures and Pearson’s chi-square tests for nominal measures. For each of these statistical analyses, and those discussed below, significance was set at *p* ≤ 0.05, and the analyses were completed using STATA 18 [15].

During this data extraction process, investigators were blinded to student identity and gender to maintain student anonymity. While certain demographic characteristics (age and race/ethnicity) were available, the inclusion of this data in the analyses discussed below was withheld to avoid identifying individual subjects and maintain student privacy protections afforded by the Family Educational Rights and Privacy Act (FERPA). Differences in these measures across cohorts were analyzed and identified when statistically significant.

To test our first hypothesis (that shortened clerkship length does not impact subject exam scores), a two-sample *t*-test comparing scores on IM and Surgery subject exams between the control and experimental groups was completed. The two-sample *t*-test assumed homogeneity of variances, with the independent variable set as the student group (pre- or post-intervention) and the dependent variable as average score on the individual NBME subject exam (IM and Surgery considered separately). We also completed one-way ANOVAs comparing exam scores across all four cohorts (academic years) for IM and Surgery. For this single-factor ANOVA model, the independent variable was set as the cohort, and the dependent variable was the average score on the individual NBME subject exam. This second analysis was performed to evaluate for any meaningful differences in cohort exam performance within the pre- and post-intervention groups that would be missed with just the pre- vs. post-intervention comparison.

While the length of the remaining year 3 core clerkships did not change, we also completed the same two-sample *t*-test and one-way ANOVA analyses on these subject exams to evaluate for any effect of shortened IM and Surgery clerkships on NBME subject exam performance in other areas. This approach of evaluating the potential effect of shortened IM and Surgery clerkships within all year 3 core clerkships was then extended through the inclusion of restricted maximum likelihood (REML) mixed-effects regression analyses that evaluated the impact of this clerkship change across all six NBME subject exam score areas. The first (of two) of these mixed-effects models included our measure of before and after the clerkship change intervention, subject exam area, and the interaction between the two as fixed effects, with the individual student set as the random effect. The second, full model also included MCAT performance and block sequence as additional covariates.

**Fig. 1:**
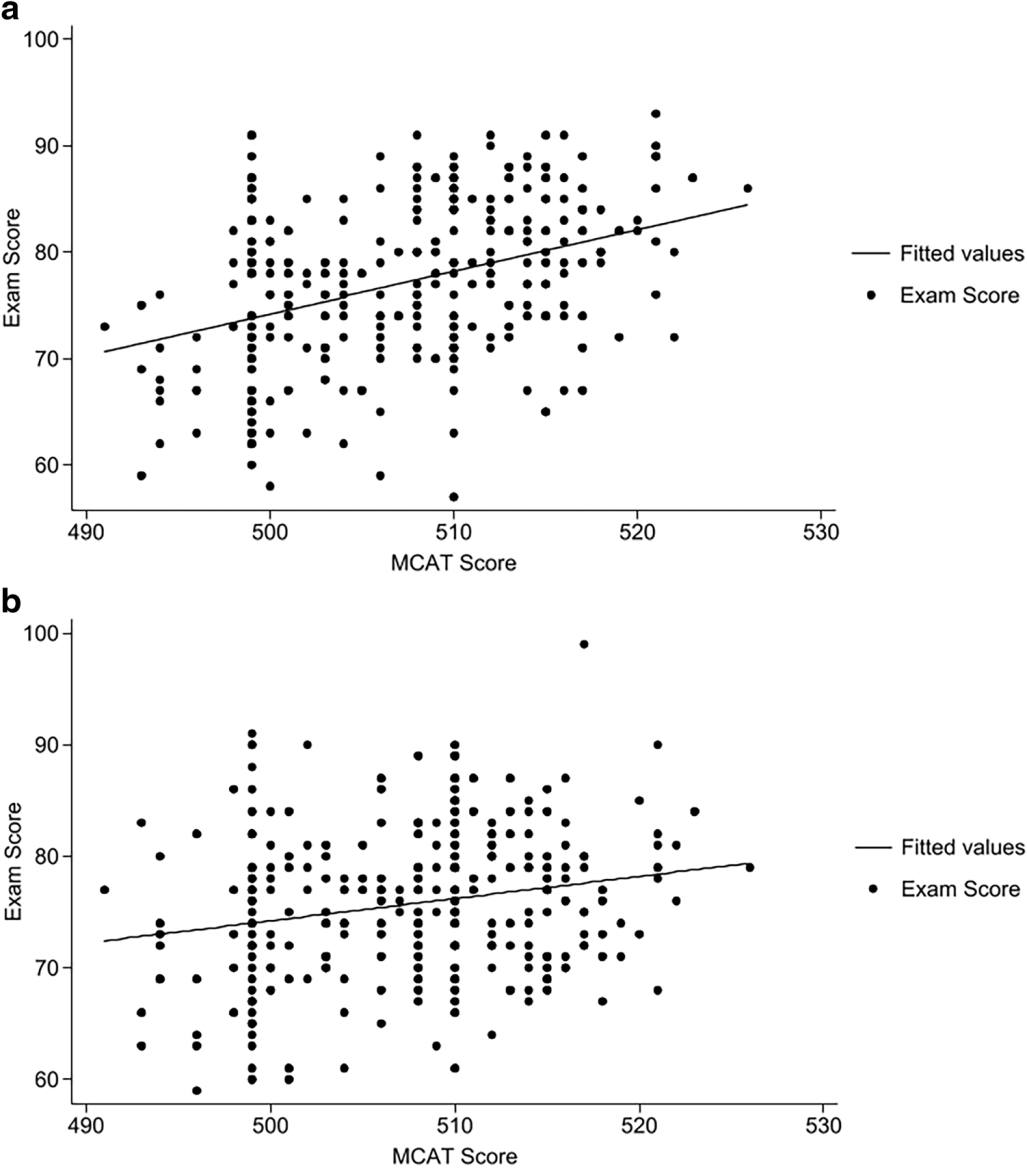
**a** Linear regression model of IM NBME subject exam score on MCAT score. **b** Linear regression model of Surgery NBME subject exam score on MCAT score

Individual student MCAT performance was an important inclusion as a covariate, as the literature demonstrates that MCAT scores are predictive of performance on USMLE and NBME exams and of year 3 grades [16–19]. MCAT scores reported on the old reporting scale (prior to April 2015) were converted to the new scoring scale via an online score converter to enable comparison across the four cohorts [20]. The correlation between MCAT score and NBME exam performance (and its relevance here) was assessed through (1) ANCOVAs evaluating the association between MCAT scores and NBME subject exam scores when factoring in differences pre- vs. post- intervention for each clerkship; and (2) subsequent regression analyses, including ultimately the full mixed-effects model discussed above. Each of these regression analyses assumed a linear relationship between MCAT performance and subject exam score, as shown in Fig. 1a and b found in the “Results” section [16–19].

Additionally, to test our secondary hypothesis (that NBME subject exam scores could be impacted by timing of the associated clerkship within the sequence of the academic year), the block sequence in which a given NBME subject exam was taken was included as a covariate as well. The correlation between this sequence measure and NBME exam performance was assessed through two-way ANOVAs evaluating the association between sequence and NBME subject exam scores when factoring in differences pre- vs. post- intervention for each clerkship and through subsequent regression analyses, which include the full mixed-effects model discussed previously. Each of these regression analyses assumed a unique, block-specific relationship with subject exam score, and that NBME exam order reflected clerkship sequence, since students take the exam at the end of the clerkship block before starting their next rotation [4].

## Results

### Impact of Clerkship Length on Subject Exam Scores

The number of student examinees in each clerkship, as well as age and MCAT characteristics for each of the four cohorts, is presented in Table 1. Race/ethnicity data is withheld to avoid identifying individual subjects. One-way ANOVA demonstrated significant differences in age across the four cohorts (*p* < 0.001), but overall, the approximately half a year age difference between the pre- and post-intervention groups was not statistically significant. Significant differences in MCAT scores across the four cohorts were seen on one-way ANOVA as well (*p* < 0.001). Most notably, the 2021–2022 academic year (AY) cohort had a higher mean MCAT score (513.8) compared with the other three cohorts (AY 2017–2018, 507.1; AY 2018–2019, 506.5; AY 2019–2020, 502.2). However, the 1-point increase in mean MCAT score between the pre- and post-intervention groups was not statistically significant in the two-sample *t*-test.

**Table 1.**
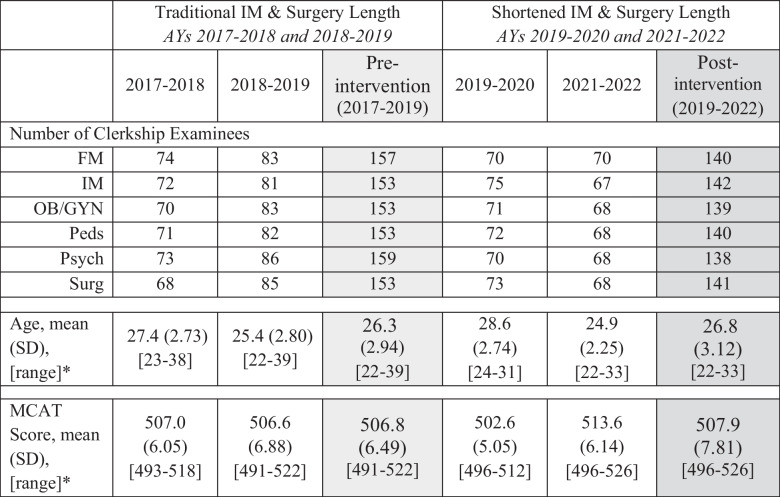
Characteristics of study population by academic year

*Abbreviations: AY*, academic year; *FM*, family medicine; *IM*, internal medicine; *OB/GYN*, obstetrics and gynecology; *Peds*, pediatrics; *Psych*, Psychiatry; *Surg*, surgery; *SD*, standard deviation; *MCAT*, Medical College Admission Test

*Statistically significant differences in age and MCAT score across the four cohorts were demonstrated on one-way ANOVA (*p*-value <0.001), but two-sample t-test showed no significant difference between the pre- and post-intervention groups. Please refer to text for details

Student NBME subject examination performance in the pre- and post-intervention time periods is summarized in Table 2. Note that while we are reporting average exam scores, analyses were performed using individual student data. When comparing the control and experimental groups through two-sample *t*-test, there was no significant difference in IM subject exam scores (*p* = 0.75). There was a statistically significant difference in IM subject exam scores across cohort years in one-way ANOVA (*p* = 0.03). Pairwise comparison of means by year revealed that this was due to a statistically significant increase in scores from 2019–2020 to 2021–2022 (within the post-intervention years only). We found no significant difference in Surgery subject exam scores between the traditional and shortened clerkship length groups on two-sample *t*-test (*p* = 0.47). We also found no statistically significant difference in Surgery exam scores across the four cohorts in one-way ANOVA (*p* = 0.55).

**Table 2.**
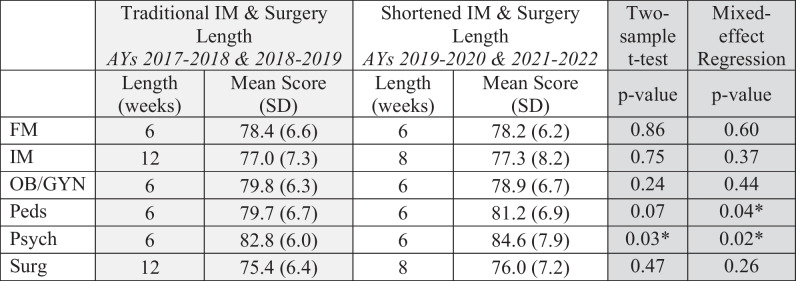
Clerkship lengths and mean NBME subject exam scores in the traditional clerkship curriculum and in the new curriculum with shortened IM and surgery clerkships

*Statistical significance driven by higher exam scores in AY 2021–2022. For psychiatry, statistically significant higher scores observed in AY 2021–2022 compared with AY 2017–2018, 2018–2019, and 2019–2020. For pediatrics, statistically significant higher scores in AY 2021–2022 compared with AY 2017–2018. Please refer to text for details

Two-sample *t*-test analyses for the remaining year 3 clerkships demonstrated no significant differences in subject exam score between the pre- and post-intervention groups for FM (*p* = 0.86), OB/GYN (*p* = 0.24), and Pediatrics (*p* = 0.07). There was a significant increase in Psychiatry subject exam scores in the post-intervention group (*p* = 0.026). One-way ANOVA examining Psychiatry exam scores across all four cohorts demonstrated that this statistical significance was driven by higher scores in the 2021–2022 cohort when compared with the first three cohorts (2017–2018, *p* = 0.002; 2018–2019, *p* = 0.015; 2019–2020, *p* = 0.006). Likewise, one-way ANOVA for Pediatrics subject exam scores demonstrated a significant difference between the 2021–2022 and 2017–2018 cohorts (*p* = 0.018), but not between the other cohorts. One-way ANOVA examining subject exam scores across the four cohorts for FM and OB/GYN revealed no significant differences (FM, *p* = 0.45; OB/GYN, *p* = 0.14).

Table 2 also displays results of the first REML mixed-effects regression analysis. This model also indicates that any difference in IM or Surgery NBME subject exam performance between the pre- and post-intervention groups was not statistically significant (IM, *p* = 0.37; Surgery,* p* = 0.26). FM and OB/GYN exam scores differences were non-significant as well. Psychiatry NBME subject exam scores remained significantly higher in the post-intervention group (*p* = 0.02) on this analysis. The post-intervention increase in Pediatrics exam scores became statistically significant in this mixed-effects model (*p* = 0.04), with pairwise comparison demonstrating this was driven by significantly higher scores in the 2021–2022 cohort compared with the 2017–2018 cohort.

Results of the second, full mixed-effects regression model investigating the impact of additional covariates (repeated measures, MCAT, block sequence) on pre- and post-intervention NBME subject exam scores are shown in Table 3. The modest increases in IM and Surgery exam scores in the post-intervention group remain, but are still nonsignificant when controlling for these additional variables. Similarly, the increases in Pediatrics and Psychiatry subject exam scores in the post-intervention group remain significant in the full mixed-effects analysis (Pediatrics, *p* = 0.036; Psychiatry, *p* = 0.012).

**Table 3:** Mixed-effects regression analysis results: includes pre-/post-intervention, individual student repeated measures, MCAT performance, and clerkship block sequence as covariates

Variable	Net change in exam score	*p*-value	Interpretation
FM	− 1.04	0.18	In the post-intervention period, scores on the FM exam decreased by 1.04 points (not statistically significant)
IM	0.97	0.23	In the post-intervention period, scores on the IM exam increased by 0.97 points (not statistically significant)
OB/GYN	− 0.40	0.62	In the post-intervention period, scores on the OB/GYN exam decreased by 0.40 points (not statistically significant)
Peds	1.66	0.036	In the post-intervention period, scores on the Peds exam increased by 1.66 points
Psych	1.99	0.012	In the post-intervention period, scores on the Psychiatry exam increased by 1.99 points
Surg	1.34	0.098	In the post-intervention period, scores on the Surgery exam increased by 1.34 points (not statistically significant)
MCAT	0.25	< 0.001	Each 1-point increase in MCAT score was significantly correlated with a 0.25 increase in NBME subject exam score (all subject areas)
Block 2	0.12	0.76	When compared with block 1, students completing a clerkship in block 2 score 0.12 points higher on all subject exams (not statistically significant)
Block 3	0.65	0.11	When compared with block 1, students completing a clerkship in block 3 score 0.65 points higher on all subject exams (not statistically significant)
Block 4	1.45	< 0.001	When compared with block 1, students completing a clerkship in block 4 score 1.45 points higher on all subject exams
Block 5	1.57	< 0.001	When compared with block 1, students completing a clerkship in block 5 score 1.57 points higher on all subject exams
Block 6	1.88	< 0.001	When compared with block 1, students completing a clerkship in block 6 score 1.88 points higher on all subject exams
Block 7	2.32	< 0.001	When compared with block 1, students completing a clerkship in block 7 score 2.32 points higher on all subject exams

### Correlation Between MCAT and NBME Subject Exam Scores

ANCOVAs were performed examining the association between MCAT performance and NBME subject exam scores and evaluating differences pre- and post-intervention. For all six clerkships, MCAT score was significantly associated with subject exam scores (FM,* p* = 0.01; IM, *p* < 0.001; OB/GYN, *p* < 0.001; Pediatrics, *p* < 0.001; Psychiatry, *p* < 0.001; Surgery, *p* < 0.001). Regression analyses demonstrated a positive linear association between individual student MCAT score and performance on each NBME subject examination, as displayed for IM and Surgery in Fig. 1a and b. For IM, each increase of 1 point on the MCAT predicted a 0.396 point increase in the subject exam score (95% confidence interval (CI), 0.28–0.51; *p* < 0.001; *R*-squared 0.14). For Surgery, each increase of 1 point on the MCAT predicted a 0.199-point increase in the subject exam score (95% CI, 0.09–0.31; *p* < 0.001; *R*-squared 0.05).

Results for REML mixed-effects regression analyses evaluating correlation between MCAT and each subject exam score while controlling for repeated student measures are shown in Table 4. For each 1-point increase in MCAT score, there was a statistically significant increase in FM, IM, Pediatrics, and Psychiatry exam scores. The largest correlation was seen with the IM subject exam (score increased by 0.266 for each 1-point increase in MCAT, *p* < 0.001). When controlling for individual students taking all subject exams with the mixed-effects model, MCAT score was no longer significantly correlated with OB/GYN and Surgery subject exam performance. Finally, the full mixed-effects regression model controlling for both repeated student measures and block sequence within the academic year demonstrated a significant correlation between MCAT and all subject exams. Each 1-point increase in MCAT score was associated with a general 0.252 increase in all NBME subject exams (*p* < 0.001, Table 3).

**Table 4:** Correlation between MCAT scores and subject exam scores: mixed-effects REML regression

	Increase in NBME subject exam score for each 1-point increase in MCAT score	*p*-value
FM	0.137	0.01
IM	0.266	< 0.001
OB/GYN	0.076	0.17
Peds	0.138	0.01
Psych	0.154	0.005
Surg	0.077	0.164

### Impact of Clerkship Sequence on NBME Subject Exam Scores

**Fig. 2:**
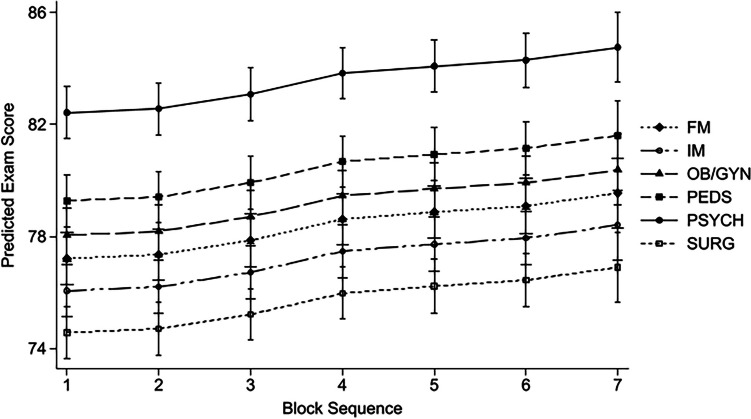
Adjusted predictions of NBME subject exam score by block sequence with 95% Cls

We hypothesized that student performance on NBME subject exams could be impacted by both the increased clinical experience gained as students progress through their clerkship year and increasing familiarity with the NBME exam experience as more exams are taken [4, 21, 22]. We therefore sought to determine if the sequence of the clinical clerkship within the academic year impacted exam performance. Two-way ANOVAs were performed evaluating the association between block sequence and individual clerkship subject exam scores. Sequence significantly impacted NBME subject exam score in the FM clerkship (*p* < 0.001) and Surgery clerkship (*p* = 0.046), with an increase of 7.28 and 3.87 points on the respective exams from the first to last sequence in the academic year. Additionally, the subsequent mixed-effects regression analysis modeling the relationship between clerkship sequence and individual student performance on the corresponding NBME subject exam demonstrated a strong positive relationship for all clerkships, as reported in Table 3 and shown in Fig. 2. A statistically significant increase above block 1 scores began in block 4 of the academic year and continued through block 7 (*p* < 0.001).

## Discussion

Our results indicate that shortening the duration of the IM and Surgery clerkships from 12 to 8 weeks has little to no impact on student performance on clinical subject exams. We found no significant changes to IM and Surgery NBME exam performance across the traditional and shortened clerkship length groups. Scores on the Psychiatry and Pediatrics subject exams increased in the post-intervention group despite no change in length in these clerkships. This suggests that some factor(s) other than clerkship length have a greater impact on student exam performance. Indeed, we found that the timing of the clerkship within the academic year was significantly correlated with exam performance for at least two clerkships (FM, Surgery). In addition, student MCAT performance independently was predictive of NBME exam score for all six clerkships. Even after controlling for clerkship sequence and MCAT score, our results did not change meaningfully — Pediatrics and Psychiatry (both unchanged in length) remained the only clerkships with a statistically significant difference in subject exam scores between pre- and post-intervention groups.

Our findings, along with previous studies by researchers such as Monrad et al., Fitz et al., and Strowd et al., suggest that student NBME exam performance, which is often used as a proxy for clinical knowledge, is largely independent of clerkship length alone [3, 11, 23]. Thus, it may be that the overall landscape of medical education is improving in quality over time, and as the quality of clinical teaching strategies improves, students may benefit from more concise clinical experiences in the long term. Our results support the growing body of evidence supporting changes in clerkship length as medical education continues to evolve. Educators should continue to weigh the optimal balance of breadth, depth, and quality of clinical experiences as they consider possible modifications to clerkship duration.

Our study is limited by several factors: we did not examine student performance on USMLE Step 2 CK or Step 3 exams, as access to this data was not granted to us under the ROME Umbrella IRB for medical student data at our institution. Additionally, we did not examine students’ self-reported clinical confidence under the different clerkship durations. We chose to study a standardized objective measure of medical knowledge (NBME exam scores), yet we recognize this may not correlate directly with clinical skill or broad clinical exposure. Additionally, like most other publications on this topic, this study was performed at a single institution, which may make generalizability more challenging. However, because of the variability in core clerkship structure across undergraduate medical education institutions and the unlikely occurrence of simultaneous clerkship reductions at multiple institutions, there is a paucity of multi-institutional research studying the impact of clerkship curricular reform on medical student knowledge and clinical preparedness. Our results may be added to existing single-center studies evaluating the impact of complementary clerkship adaptations, such as reduction in length of a single clerkship or reduction in length of all core clerkships while simultaneously making other curricular changes. Our findings add to the current literature by evaluating the impact of shortened duration in two core clerkships without other curricular changes and by controlling for potential confounding variables.

Clerkships are a foundational aspect of medical education, representing the first extended opportunity for students to learn and practice clinical skills while caring for patients. These experiences are vital for forming a foundation that can be expanded upon during residency. The quantity of these experiences must be balanced against the quality each can provide. Our research indicates that shortening the clerkship experience may allow for efficiency and greater personalization in clinical training without adverse effects on knowledge acquisition. Increased efficiency and quality in clerkship training would allow for both better retention of medical knowledge and time for students to explore additional clinical opportunities outside of the traditional core clerkships. Additional research to determine the impact of active learning interventions on a shortened clerkship structure will further add to our knowledge. Larger scale studies, while challenging to perform and analyze due to nuanced differences across multiple institutions’ clinical curricula and experiences, will advance broader understanding of the factors most instrumental in determining student success.

## Supplementary Information

Below is the link to the electronic supplementary material.

Supplementary file1 (PDF 178 KB)

## Data Availability

The datasets that support the findings of this study are available from the corresponding author (CB) upon reasonable request. The data are not publicly available due to privacy restrictions.
