# Supplementary material for: Impact of Clerkship Length and Sequence on NBME Subject Exam Performance
Source: Med Sci Educ. 2025 Feb 17;35(3):1313–22. doi: 10.1007/s40670-025-02305-y (PMC12228629; doi:10.1007/s40670-025-02305-y)

Supplemental Materials

Appendix 1 Comparison of Traditional and New Clinical Curricula

Course	Traditional* Curriculum:<br>through AY 2018-2019	New** Curriculum:<br>began in AY 2019-2020
Family Medicine Clerkship	Year 3<br>6 weeks at one clinical site	Year 3<br>6 weeks at one clinical site
Internal Medicine Clerkship	Year 3<br>12 weeks across three clinical sites	**Year 3**<br>**8 weeks across two clinical sites**
Neurology Clerkship	Year 4<br>4 weeks at one clinical site	**Year 3**<br>**4 weeks at one clinical site**
Obstetrics & Gynecology Clerkship	Year 3<br>6 weeks at one clinical site	Year 3<br>6 weeks at one clinical site
Pediatric Clerkship	Year 3<br>6 weeks across two clinical sites	Year 3<br>6 weeks across two clinical sites
Psychiatry Clerkship	Year 3<br>6 weeks at one clinical site	Year 3<br>6 weeks at one clinical site
Surgery Clerkship	Year 3<br>12 weeks across three clinical sites	**Year 3**<br>**8 weeks across two clinical sites**
Elective Rotation(s)	Five rotations total (20 weeks); all electives taken in Year 4	**Six rotations total (24 weeks); one 4-week elective taken in Year 3 and the remainder occur in Year 4**
Emergency Medicine Clerkship	Year 4<br>4 weeks at one clinical site	Year 4<br>4 weeks at one clinical site
Selective: Sub-Internship	Year 4<br>4 weeks at one clinical site	Year 4<br>4 weeks at one clinical site
Selective: Critical Care	Year 4<br>4 weeks at one clinical site	Year 4<br>4 weeks at one clinical site
Longitudinal Patient Care Course	Longitudinal course spanning Years 3 and 4.<br>Students meet in interprofessional teams with their community health mentor every 6 weeks	Longitudinal course spanning Years 3 and 4.<br>Students meet in interprofessional teams with their community health mentor every 6 weeks

*Traditional curriculum refers to academic years before 2019-2020.

**New curriculum refers to academic year 2019-2020 and beyond.
The core clerkships provide a mix of inpatient and ambulatory education in their respective specialties. Elective rotations allow for personalization of the clinical curriculum, through which students gain exposure to their intended career specialty, other relevant specialties, clinical research, etc. Selective rotations include a Core Sub-Internship and a Critical Care rotation; these may be taken in one of several core specialties and are intensive experiences to prepare students for residency. The Longitudinal Patient Care Course (LPC) provides interprofessional education; medical students work collaboratively with Physical Therapy and Occupational Therapy students to meet with and follow an adult with a chronic medical condition who serves as the students' community health mentor.

Appendix 2 Color Version of Figure 2, Adjusted predictions of NBME subject exam score by block sequence with 95% CIs

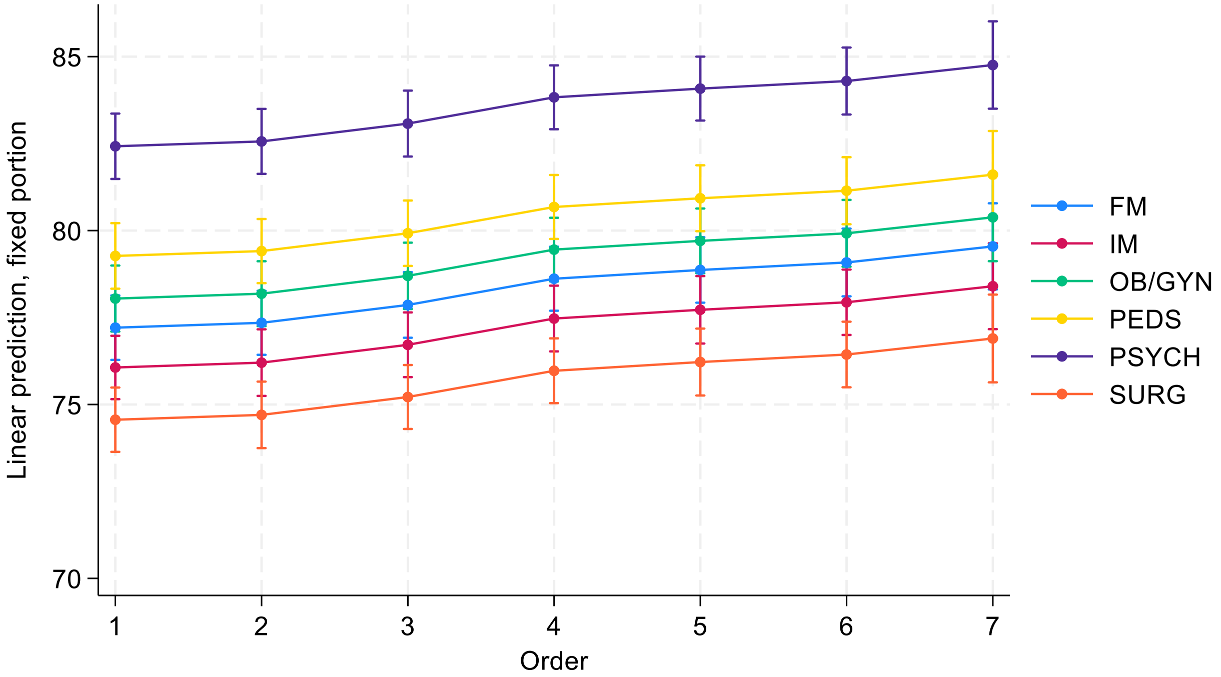

Supplement: Supplementary file 1 — Supplementary file1 (PDF 178 KB) [file 40670_2025_2305_MOESM1_ESM.pdf]
